# Mutational signatures among young-onset testicular cancers

**DOI:** 10.1186/s12920-021-01121-8

**Published:** 2021-11-24

**Authors:** Nicole E. Mealey, Dylan E. O’Sullivan, Cheryl E. Peters, Daniel Y. C. Heng, Darren R. Brenner

**Affiliations:** 1 Department of Oncology, Cumming School of Medicine, University of Calgary, Room 382B, Heritage Medical Research Building, 3310 Hospital Drive NW, Calgary, AB T2N 4N1 Canada; 2 Department of Community Health Sciences, Cumming School of Medicine, University of Calgary, Calgary, AB Canada; 3 Department of Cancer Epidemiology and Prevention Research, Alberta Health Services, CancerControl Alberta, Calgary, AB Canada; 4 CAREX Canada, Simon Fraser University, Vancouver, BC Canada; 5 Department of Internal Medicine, Medical Oncology, Alberta Health Services, Calgary, AB Canada

**Keywords:** Mutational signatures, Testicular neoplasms, Young-onset, Genomics, Somatic mutations

## Abstract

**Background:**

Incidence of testicular cancer is highest among young adults and has been increasing dramatically for men born since 1945. This study aimed to elucidate the factors driving this trend by investigating differences in mutational signatures by age of onset.

**Methods:**

We retrieved somatic variant and clinical data pertaining to 135 testicular tumors from The Cancer Genome Atlas. We compared mutational load, prevalence of specific mutated genes, mutation types, and mutational signatures between age of onset groups (< 30 years, 30–39 years, ≥ 40 years) after adjusting for subtype. A recursively partitioned mixture model was utilized to characterize combinations of signatures among the young-onset cases.

**Results:**

Mutational load was significantly higher among older-onset tumors (*p* < 0.05). There were no highly prevalent driver mutations among young-onset tumors. Mutated genes and types of nucleotide mutations were not significantly different by age group (*p* > 0.05). Signatures 1, 8 and 29 were more common among young-onset tumors, while signatures 11 and 16 had higher prevalence among older-onset tumors (*p* < 0.05). Among young-onset tumors, clustering of signatures resulted in four distinct tumor classes.

**Conclusions:**

Signature contributions differ by age with signatures 1, 8 and 29 were more common among younger-onset tumors. While these signatures are connected with endogenous deamination of 5-methylcytosine, late replication errors and chewing tobacco, respectively, additional research is needed to further elucidate the etiology of young-onset testicular cancer. Large studies of mutational signatures among young-onset patients are required to understand epidemiologic trends as well as inform targeted prevention and treatment strategies.

**Supplementary Information:**

The online version contains supplementary material available at 10.1186/s12920-021-01121-8.

## Background

### Epidemiology of testicular cancer

There were an estimated 8720 cases of testicular cancer diagnosed and 380 deaths due to testicular cancer in the United States in 2016 [1]. In Canada, the projected incidence and mortality was 1100 and 45 cases respectively in 2017 [2]. Testicular cancer accounts for 1% of new cancer diagnoses among men in the United States and Canada [2–4]. Age standardized incidence rates around the world were between 1 and 9.2 per 100,000 in 2004 [3]. Worldwide incidence of testicular cancer doubled between 1964 and 2004, and has been increasing since the mid 1900’s in North America and northern Europe [3]. In Canada, a recent study showed an increasing risk of testicular cancer for men born after 1945 [5]. The increase in incidence in Canada was largest among men 20–29 and a slightly lower increase was observed among men 30–39 during the same timeframe. Between 1992 and 2009, testicular cancer incidence increased by an average of 1.1% annually in the United States and Europe [6].

Incidence of testicular cancer peaks among men at age 35 [3, 5]. Among Canadians aged 15–29, testicular cancer makes up 14% of cancer diagnoses, making it the second most common new cancer diagnosis in this age group [2]. Young cancer patients (ages 15–39) fall between pediatric oncology and medical oncology targeted at older adults, and face particular challenges obtaining treatment [7].

Nearly all testicular cancers are germ-cell neoplasms [3]. Of these, about half are seminomas and the other half are non-seminomas [3, 8, 9]. This histological distinction is highly relevant to tumor etiology and treatment, as non-seminoma tumors are more likely to metastasize [3]. The distribution of histology has been shown to vary by age, where the transition of a germ cell neoplasia in situ (GCNIS) to a testicular cancer is more likely to result in a nonseminomatous tumor if it occurs at a younger age.

The reasons for the rising incidence in testicular cancer have not been fully elucidated. There are few established risk factors for testicular cancer, and these include preterm birth and cryptorchidism [3, 10]. In utero exposure to synthetic estrogen diethylstilbestrol (DES) has been shown to be associated with a threefold increase in the risk of testicular cancer in a recent meta-analysis [11], but DES prescriptions ceased in the early 1970s while testicular cancer incidence has continued to rise [3]. GCNIS is believed to precede testicular cancer in almost all cases [3, 9]. A link between maternal smoking and testicular cancer has not been consistently supported [3, 12, 13]. Heavy or long-term cigarette smoking is associated with increased risk of testicular cancer, and smoking status is related to more aggressive tumors [14–16]. Similarly, heavy or long-term cannabis use is associated with increased risk of testicular cancer, primarily for nonseminoma subtype, and particularly if it begins before age 18 [16–18]. Other proposed risk factors include lifestyle changes accompanying greater prosperity, including changes to diet (e.g. high fat and dairy consumption), and increased sedentary behaviour.

### Genomics and understanding etiology

Processes of DNA damage (both exogenous and endogenous), as well as DNA repair leave their imprint on the genomes of cancerous cells. By studying the landscape of somatic mutations in these cells, we may identify the mutational forces driving oncogenesis. The etiologies behind some mutational signatures are already known, and understanding mutational signatures may help to elucidate the etiologies of testicular cancer. Mutational signatures are distinct patterns of somatic mutations that arise as a result of errors in DNA replication, faulty repair pathways, or exposure to exogenous mutagens [19]. They can be extracted from a tumor’s variation and examined to elucidate possible factors in the tumor’s etiology. In particular, examining differences in the mutational signatures by age of onset may help to identify particular emerging exposures since cancer risk and exposure latency periods vary by age. This is important, since testicular cancer is a relatively rare cancer, which makes it difficult to study in a timely manner in a traditional epidemiologic study setting. In addition, studying the mutational landscape could help understand the forces behind the increasing rates among young people as well as inform treatment. For instance, mutational load has been shown to influence response to immunotherapy [20].

In a previous analysis of molecular alterations of testicular cancer tumors in The Cancer Genome Atlas (TCGA), the authors identified that somatic mutations of three genes—*KIT*, *KRAS*, and *NRAS—*were commonly observed in testicular cancers [8]. In addition, they observed that the most frequent type of base alteration was a cytosine to thymine and that the most common mutational signature correlates with the COSMIC signature 1, which is the result of the accumulation of 5-methylcytosine deamination events. While highly informative in their presentation of data, the authors have not presented a differential analysis of genomic alterations by age at onset, which would provide some additional information into the increasing trends among young-onset cases.

To our knowledge no previous study has examined in detail the differences in the mutational landscape of young- and older-onset testicular cancers. The objective of this study is therefore to determine if there are differences in the mutational load, prevalence of specific mutations, and/or mutational signatures between young- and older-onset testicular cancer patients.

## Methods

Clinical and somatic mutation data were downloaded from TCGA [21] using the Genomic Data Commons Data Transfer Tool [22] on May 17, 2019. Cases were included if the primary site was the testis, and both Variant Call Format (VCF) files and clinical data files were available. VCF files were filtered to remove insertions and deletions, and single nucleotide variants (SNVs) that were flagged as problematic by the MuTect2 pipeline [23]. Cases were divided into two young-onset groups: diagnosed before age 30 and diagnosed between ages 30 and 39; and an older-onset group (diagnosed at or after age 40). The threshold chosen to divide age of onset groups matches the National Cancer Institute’s definition of “young people” [24] and corresponds to age groups with different trends in incidence (20–29 with a greater increase in recent years compared to 30–39). Given that the increasing incidence of testicular cancer is occurring in the 20–29 and 30–39 age groups, we wanted to determine if there were molecular differences for those age groups compared to the 40 + age group. In addition, age was modeled as a continuous variable in each analysis to further elucidate the effect of age. We also separated samples into seminoma and non-seminoma histological types. Tumors with mixed histology were classified as non-seminoma.

### Mutational load

We examined the number of SNVs present in each sample. We compared the distribution of mutational load between young- and older-onset groups using linear regression. The distributions were right-skewed, so the natural log-transformed mutational loads were compared. We also examined mutational load between age of onset groups adjusted for histologic subtype. A 0.05 level of significance was used throughout this study.

### Mutated genes

Shen et al. noted that the *KIT*, *NRAS*, *KRAS* genes were significantly mutated among this sample of testicular tumors [8]. Multiple *PIK3CA* and *PIK3CD* mutations were also observed in this sample. We analyzed the prevalence of mutations within these genes of interest [25], then compared the prevalence between age of onset groups using logistic regression, adjusted for histologic subtype.

### Mutation types

We examined the number and proportion of the six possible nucleotide alterations—C > A, C > G, C > T, T > A, T > C, and T > G—in each age of onset group. We tested for differences in the proportion of each mutation type between age groups with linear regression models adjusted for histologic subtype.

### Mutational signatures

Identifying mutational signatures in samples with a low number of SNVs is susceptible to higher error, particularly for signatures that lack strong peaks [26]. To mitigate this, we excluded cases with fewer than 40 SNVs after filtering [27]. The R package “SomaticSignatures” [28] was used for de novo extraction of signatures [29]. The “assessNumberSignatures” method was used to determine the number of signatures “r” to use for de novo extraction, then these signatures were visually inspected for resemblance to canonical signatures from the Catalogue of Somatic Mutations In Cancer (COSMIC) [19]. Where the ideal “r” value was unclear we looked at multiple, and used a combination to refine a list of COSMIC signatures that are likely present in our sample. Additional file 1: Fig. S1, Additional file 2: Fig. S2, Additional file 3: Fig. S3, Additional file 4: Fig. S4 and Additional file 5: Fig. S5 depict the process and results of our selection of the ideal “r” value. We used the R package “deconstructSigs” [26] to calculate contributions of this refined list of COSMIC signatures towards the mutational spectrum of our tumor samples. The prevalence of the list of COSMIC signatures were compared between age groups overall using a logistic regression adjusted for histologic subtype. Signatures were determined to be present in a sample by the “deconstructSigs” algorithm if they were calculated to have a contribution > 6% towards the sample’s mutational spectrum.

### Clustering of mutational signature contributions

To characterize combinations of mutational signature contributions in young-onset testicular cases, we employed a recursively partitioned mixture model (RPMM) clustering analysis using the R package “RPMM” [30] on common mutational signatures among this group (prevalence > 25%). In order for the signatures to be on the same scale, each signature was normalized before the RPMM was conducted. Differences in class membership by age (20–29 vs. 30–39) and subtype were tested using Fisher’s exact permutation tests were performed.

## Results

### Description of data

Data from TCGA included 150 cases of testicular cancer. Most participants were Caucasian (89%). All cases had VCF files available, and 134 had clinical data files available. Of these, 59 were diagnosed between ages 20 and 29, 54 were diagnosed between ages 30 and 39, and 21 were diagnosed at age 40 or later. After filtering variant calls, the median number of SNVs was 52. We excluded 33 cases that had fewer than 40 filtered SNVs, leaving 101 cases with at least 40 SNVs. Of the remaining cases, there were 40, 41, and 20 cases in the 20–29, 30–39, and 40 or over age of onset groups. For the analyses by histologic subtype, one cases in the 30–39 age group was removed because tumor type data was unavailable. The 100 remaining samples were comprised of 44 seminomas and 56 non-seminomas.

### Mutational load

The median number of SNVs observed within tumors among the 20–29, 30–29, and > 40 age of onset groups were 50, 51 and 65, respectively. The difference in mutational load between age groups was statistically significant for both the 20–29 age group (*p* = 0.019) and the 30–39 age group (*p* = 0.019) compared to cases diagnosed after age 40. This difference was still significant after adjusting for tumor subtype for both the 20–29 (*p* = 0.006) and 30–39 (*p* = 0.012) age groups. The effect estimate for the youngest age group was − 0.36 (95% Confidence Interval (CI) − 0.61 to − 0.11), and for the middle age group was − 0.32 (95% CI − 0.58 to − 0.071). Age as a continuous variable was also significantly associated with mutational load (*p* = 0.002) with an effect estimate of 0.015 (95% CI 0.005 to 0.024) per increase in one year of age.

### Mutated genes

The *KIT* gene had the highest overall prevalence of mutations, with mutations present in 16% of tumors (Table 1). *KRAS* mutations were present in 10% of testicular tumors, while fewer than 5% of samples contained mutations in *NRAS*, *PIK3CD*, and *PIK3CA* genes. About a third of older-onset tumors contained KIT mutations, which is a higher proportion than among young-onset tumors (12% and 15% in the 20–29 and 30–39 age of onset groups respectively). The difference between the 20–29 and 40 and over age groups was significant (*p* = 0.032), but became non-significant when adjusted for tumor type (*p* = 0.25) Both *KIT* and *NRAS* mutations were about twice as common among older-onset tumors than the two young-onset groups. All mutations in these genes of interest were in seminomas, except for one *PIK3CD* mutation found in a non-seminoma. The only gene of interest that was more often mutated among young-onset tumors was *PIK3CA*. However, only 3 samples had a *PIK3CA* mutation, 1 in the 20–29 group, and 2 in the 30–39 group, while there were none among older-onset samples.

**Table 1:** Prevalence of gene mutations

Gene	20–29	30–39	> 40	Total	*p*-value (20–29 vs. > 40)	*p*-value (30–39 vs. > 40)	*p*-value (continuous age)
KIT	7 (12%)	8 (15%)	7 (33%)	22 (16%)	0.25	0.093	0.20
KRAS	4 (7%)	7 (13%)	3 (14%)	14 (10%)	0.65	0.99	0.55
NRAS	2 (3%)	2 (4%)	2 (10%)	6 (4%)	0.65	0.19	0.47
PIK3CD	1 (2%)	3 (6%)	1 (5%)	5 (4%)	0.62	0.84	0.66
PIK3CA	1 (2%)	2 (4%)	0 (0%)	3 (2%)	NA	NA	0.95

Prevalence of somatic mutations in genes of interest among testicular tumors diagnosed at ages 20–29 (n = 59), 30–39 (n = 54), age 40 or older (n = 21), and at any age (n = 134). Entries are number (proportion) of cases in each age of onset group with each gene present. Differences in gene mutations by age at onset groups were tested using a logistic regression model * indicates statistical significance at a .05 level

### Mutation types

The most common type of nucleotide alteration for all three age of onset groups was C > T, accounting for over one third of alterations in each group (Table 2). T > A and T > G mutations accounted for fewer than 10% of mutations in each group. No significant differences in the proportion of mutation types between groups was observed.

**Table 2:** Types of nucleotide alterations

Gene	20–29	30–39	> 40	*p*-value (20–29 vs. > 40)	*p*-value (30–39 vs. > 40)	*p*-value (continuous age)
C > A	1510 (21%)	1268 (21%)	582 (18%)	0.085	0.10	0.18
C > G	896 (12%)	742 (12%)	460 (15%)	0.15	0.22	0.18
C > T	2712 (37%)	2220 (37%)	1120 (36%)	0.94	0.76	0.80
T > A	566 (8%)	440 (7%)	240 (8%)	0.79	0.86	0.79
T > C	1182 (16%)	986 (16%)	518 (16%)	0.64	0.82	0.90
T > G	494 (7%)	352 (6%)	228 (7%)	0.54	0.12	0.82

Prevalence of somatic nucleotide alterations among testicular tumors diagnosed at ages 20–29 (n = 59), 30–39 (n = 54), and age 40 or older (n = 21). Entries are number (proportion) of mutations type in each age of onset group. Differences in types of nucleotide laterations by age at onset groups were tested using a logistic regression model * indicates statistical significance at a .05 level

### Mutational signatures

**Fig. 1:**
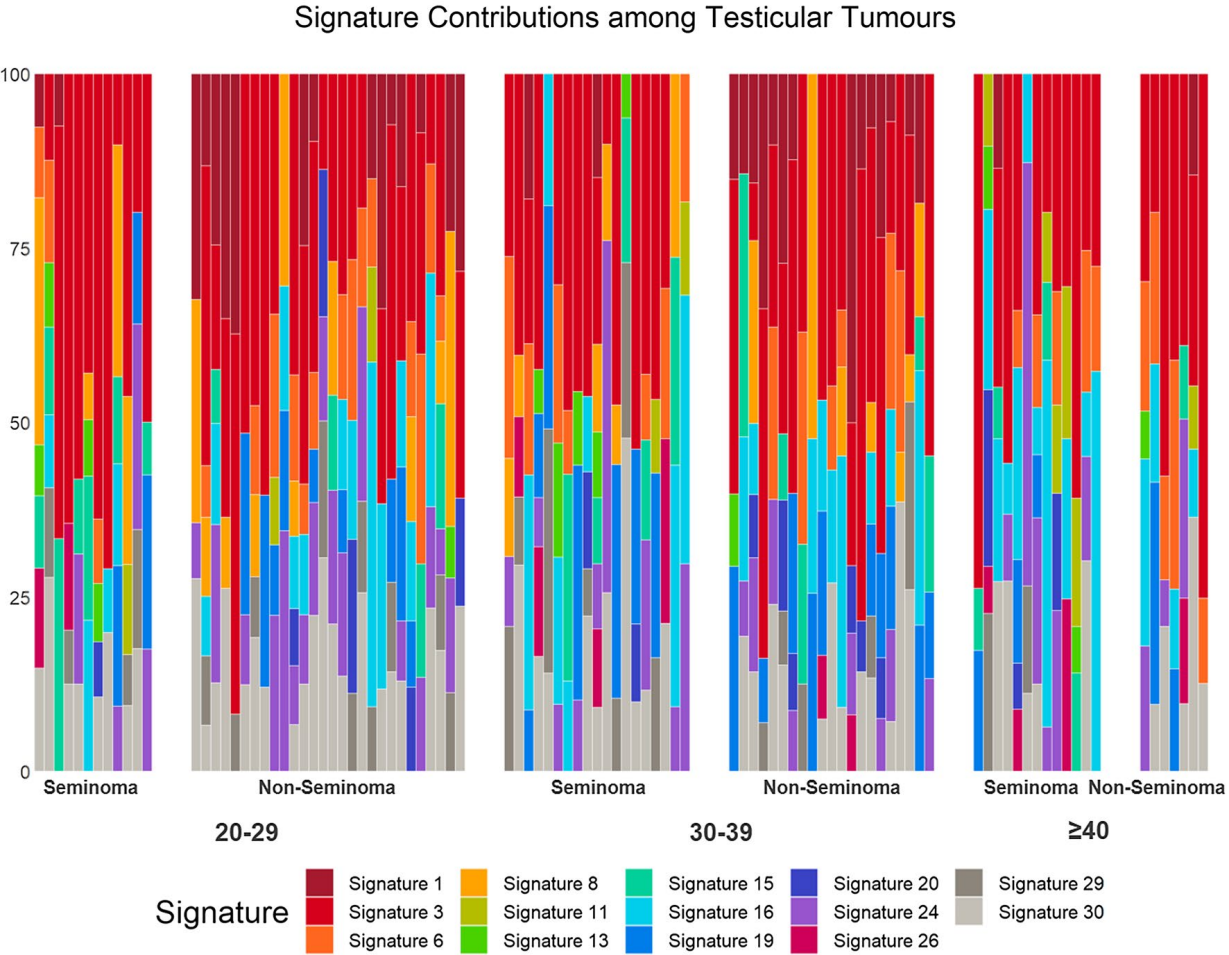
Contributions of mutational signatures to mutational spectra of testicular cancer. A set of COSMIC signatures were extracted using R package “deconstructSigs”. Samples are divided into age of onset groups 20–29 (n = 40), 30–39 (n = 40), and ≥ 40 (n = 20), and subdivided into seminoma and non-seminoma groups. Within subgroups, samples are sorted by increasing age of onset

Signature 1 was more prevalent among non-seminoma tumors compared to seminoma tumors (*p* < 0.001), while signatures 13, 15, and 26 were more common among seminoma tumors (*p* = 0.006, 0.032, and 0.048 respectively) (Additional file 6: Table S1). The difference between the 20–29 and ≥ 40 age groups for signatures 1 and 11 was significant before adjusting for tumor type (*p* = 0.027 and 0.031, respectively) but after adjustment was no longer statistically significant (*p* = 0.12 and 0.10 respectively) (Table 3). When evaluated with age as a continuous variable, the difference in signature 11 was significant (*p* = 0.010) while it was not in signature 1 (*p* = 0.14). Signature 1 was more common among tumors diagnosed in the 30–39 age group (39%) compared to older-onset tumors (10%, *p* = 0.047). Signature 29 was present in 35% of tumors diagnosed in the 20–29 age group, which was significantly greater than the 10% of tumors diagnosed at age 40 or over (*p* = 0.039). Conversely, signatures 11 and 16 were more prevalent in the older age of onset group. This difference was significant when compared to both younger groups for signature 16, though it was not upheld when examining age continuously. Signature 11 was significantly more common in the older age group compared to the 30–39 age group, but not the 20–29 age group. When analyzing age continuously, the prevalence of signature 29 was significantly higher at younger ages of onset (*p* = 0.036). Of note, this difference was also significant for signature 8 (*p* = 0.020). The contributions of mutational signatures to tumors within each tumor type and age group are visualized in Fig. 1.

**Table 3:** Prevalence of mutational signatures

Signature	20–29	30–39	> 40	*p*-value (20–29 vs. > 40)	*p*-value (30–39 vs. > 40)	*p*-value (continuous age)
Signature 1	16 (40%)	16 (39%)	2 (10%)	0.12	0.047*	0.14
Signature 3	35 (88%)	34 (83%)	18 (90%)	0.69	0.41	0.54
Signature 6	17 (42%)	13 (32%)	10 (50%)	0.40	0.15	0.55
Signature 8	14 (35%)	14 (34%)	0 (0%)	NA	NA	0.020*
Signature 11	3 (8%)	2 (5%)	6 (30%)	0.10	0.026*	0.010*
Signature 13	5 (12%)	6 (15%)	3 (15%)	0.64	0.73	0.29
Signature 15	11 (28%)	11 (27%)	5 (25%)	0.43	0.78	0.51
Signature 16	16 (40%)	16 (39%)	14 (70%)	0.034*	0.032*	0.13
Signature 19	12 (30%)	17 (41%)	5 (25%)	0.85	0.23	0.80
Signature 20	6 (15%)	8 (20%)	3 (15%)	0.83	0.73	0.90
Signature 24	21 (52%)	17 (41%)	9 (45%)	0.69	0.80	0.55
Signature 26	2 (5%)	6 (15%)	4 (20%)	0.22	0.84	0.49
Signature 29	14 (35%)	12 (29%)	2 (10%)	0.039*	0.085	0.036*
Signature 30	28 (70%)	23 (56%)	10 (50%)	0.23	0.82	0.32

Prevalence of COSMIC mutational signatures among testicular tumors diagnosed at ages 20–29 (n = 40), 30–39 (n = 41), and age 40 or older (n = 20). Entries are number (proportion) of cases with a signature present above a threshold of 6% in each age of onset group. Differences in signatures by age at onset groups were tested using a logistic regression model * indicates statistical significance at a .05 level

### Clustering of mutational signature contributions

**Fig. 2:**
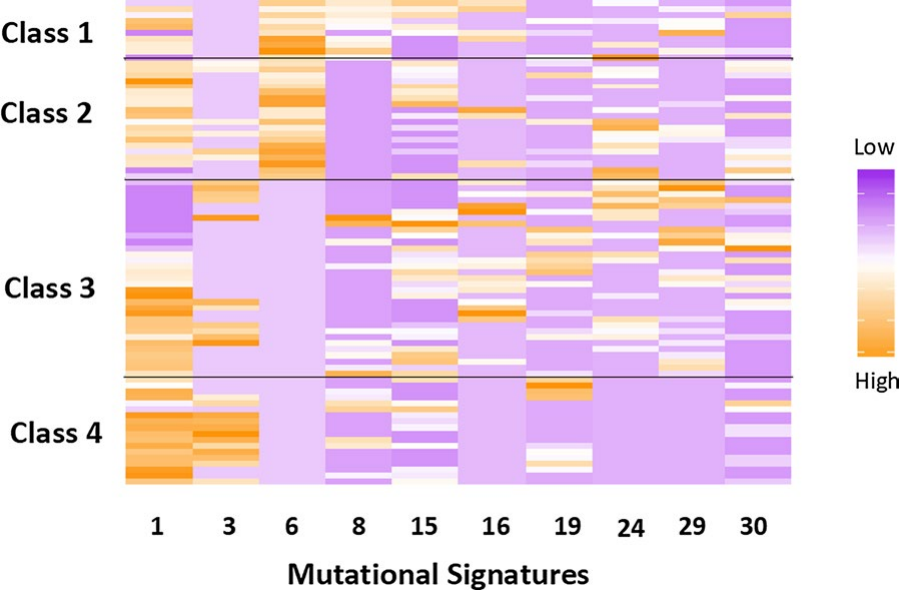
Recursively partitioned mixture model of common mutational signature contributions among testicular cancer patients under the age of 40

Among young-onset testicular cancer cases the RPMM generated four distinct classes (Fig. 2). Among the four classes, the first class (n = 10) had large contributions from signatures 1, 6, and 8, while the second class (n = 20) had large contributions from signatures 1 and 6, but no contribution from signature 8. The third class (n = 33) had a large contribution signature 1, no contribution from signature 6 and small contributions from several other signatures. Finally, class four (n = 18) was composed of primarily signature 1 and 3. There was no statistical evidence of differences in class membership by subtype (*p* = 0.69) and the young age groups (*p* = 0.24). However, 80% of class one was composed of patients < 29 years old.

## Discussion

In this study we observed that mutational load was higher among older-onset tumors. We observed statistically significant differences in the prevalence of signatures 1, 11, 16, and 29 between age of onset groups. Signatures 1 and 29 were more common among younger-onset groups than older-onset tumors. In contrast, signatures 11 and 16 had higher prevalence among older-onset tumors. Among young-onset tumors, clustering of signatures resulted in four classes with contributions from different signatures.

Our observations that older-onset tumors had a significantly higher mutational load than young-onset tumors likely resulted from longer lifetime accumulation of insults to the DNA of older patients. The majority of mutations we observed within genes of interest were in seminomatous tumors. Somewhat surprisingly, there were no highly prevalent driver mutations in genes among young-onset tumors. This indicates that some other carcinogenic process may be driving young-onset tumorigenesis. One alternative is mutations outside the exome, which would not be detected by our methods, but could alter cellular functioning. Another possibility is differences in or environmental impacts to the epigenome, which can influence gene expression and subsequent cellular functions. Shen et al. described a subset of seminomatous testicular tumors that were characterized by *KIT* mutations and other molecular differences [8]. These tumors had lower methylation at CpG islands, which reduced mutations contributing to signature 1. However, there were no statistically significant differences in mutated gene prevalence between age of onset groups for *KIT* or the other four genes of interest.

When examining types of nucleotide alterations, we did not observe any significant difference between young- and older-onset tumors. It was not until they were decomposed into the more complex mutational signatures that observable differences arose. Signatures 1 and 29 were more common among the 30–39 and 20–29 age of onset tumors respectively, compared to older-onset tumors. Signature 1 arises as a result of spontaneous deamination of 5-methylcytosine and is typically correlated with age [19, 31], making it unusual to observe higher prevalence of signature 1 among younger-onset tumors. This result may be artifactual, or may represent some unknown pressure that is increasing the rate of this endogenous process among this group of tumors. Signature 29 is associated with chewing tobacco and has been observed among gingivo-buccal oral squamous cell carcinoma [19, 32]. To our knowledge there have been no previous studies examining an association between smokeless tobacco and the risk of testicular cancer. Indeed, a recent systematic review on the relationship of smokeless tobacco and cancer did not include testicular cancer as one of the 20 potential outcomes and did not identify any studies examining testicular cancer [33]. Among non-TCGA samples with > 50 mutations, another study observed that 5 of 16 patients (31.3%) had greater than a 10% contribution from signature 29 [34]. More research on the potential relationship of smokeless tobacco products, as well as e-cigarettes with testicular cancer are required. Given the documented relationship of smoking with testicular cancer, the lack of signature 4 in this study is surprising but could be due to the small select sample, which might include few smokers.

The lack of cases in the older age of onset group with contributions from signature 8 precluded a categorical comparison, but the continuous analysis showed that even among tumors diagnosed before age 40, signature 8 was more likely to play a role at younger ages of onset. There is some evidence supporting that signature 8 arises from replication errors that go unrepaired as cancer cells are rapidly dividing [35]. Given that putative mutations in DNA repair genes have not been previously reported for testicular cancer [8, 34], we hypothesize that epigenetic alterations, such as aberrant DNA methylation, are responsible for deficiencies in DNA repair. Indeed, large differences in DNA methylation, including DNA repair genes have been observed across histologic subtypes of testicular cancer [8, 34]. Given that a major part of male germ cell development occurs during prenatal period, novel prenatal exposures may lead to epigenetic disruption that manifests into cancer during early adulthood. Future studies should examine age-specific patterns in DNA methylation of testicular cancer and potential related exposures, particularly novel prenatal exposures, to further elucidate mechanisms responsible for the increased incidence among younger age groups.

Two signatures were observed to be more common among the older-onset group relative to the 30–39 group for signature 11, and relative to both younger-onset groups for signature 16. Signature 11 is associated with exposure to alkylating agents [19, 36]. Our findings suggest there may be some environmental exposure to alkylating agents leading to this higher prevalence of signature 11 among older-onset testicular tumors. The etiology of signature 16 is unknown [19], so further research will be necessary to elucidate its significantly higher prevalence among older-onset testicular tumors.

Within the young-onset group, RPMM analysis of the mutational signatures present revealed four distinct groups. Spontaneous deamination is linked with signature one, which was consistent across all four classes. The first two classes also had signature 6, and were distinguished by the presence of signature 8 in class 1. Signature 6 is related to defective mismatch repair and microsatellite instability, and the etiology of signature 8 is unknown [19]. Class four had signature 3, which is connected with missing double-strand break repair by homologous recombination. The four classes identified in our analysis may have distinct etiologies which should be explored in future studies.

We attempted to limit mutational signature bleeding in this study by conducting de novo signature extraction followed by linking these to COSMIC signatures based on pattern similarity. The most common signatures observed in the study sample were signatures 1 and 3, which is consistent with a previous study on testicular cancer [34]. Less common signatures observed in this study have also been previously reported [34]. While signature bleeding cannot be completely ruled out from this study, it is unlikely that signature bleeding would influence the age-specific results unless there was signature contamination of an age-related signature. It is more likely that bleeding would occur non-differentially across age groups. For instance, signature 3, 5, and 8 are flat signatures that resemble each other [37]. It is therefore possible that the predominance of signature 8 among young-onset patients was due to bleeding from signature 5, which did not contribute significantly in this analysis. However, signature 5 is related to aging and therefore bleeding from signature 5 would likely contribute to the opposite finding (predominance of signature 8 in older-onset patients) of this study. Nevertheless, studies examining age-specific mutational signatures of testicular cancer in an independent sample are required to confirm these findings.

In this study, we observed differences in mutational signatures which suggests a different etiology for young-onset testicular cancers. Signatures 1 and 29 were more prevalent among young-onset tumors, while signatures 11 and 16 were more common in the older-onset group. The lack of a highly prevalent driver mutations among young-onset testicular tumors indicates that an alternative process is driving carcinogenesis. More research is needed to understand the driving factors behind age of onset-related differences. Examining larger sample sizes, and additional genomic and epigenomic alterations will provide a stronger understanding of etiological differences by age of onset. Further research in populations with detailed exposure measures will allow us to explore whether the differential prevalence in signatures 1, 11, 16 and 29 across age at onset groups are related to particular environmental factors or behaviours.

COSMIC recently released version 3 of their mutational signatures, which includes signatures comprised of doublet base substitutions and small insertions and deletions as well as single base substitutions. Further studies should investigate the processes that lead to signature 16 mutations, as this may be important for understanding young-onset testicular cancer etiology.

## Conclusion

The lack of highly prevalent driver mutations among young-onset testicular tumors suggests that alternative factors, such as epigenetics may be driving tumorigenesis among young patients. Signatures 11 and 16 were more prevalent among older-onset testicular tumors, while signatures 1, 8 and 29 were more prevalent among young-onset tumors. A larger sample size is recommended to clarify associations between mutational signatures and age of onset of testicular cancer. Further research is underway to compare prevalence of COSMIC’s version 3 signatures between age of onset groups.

## Supplementary Information

**Additional file 1: Figure S1**. The residual sum of squares (RSS) and explained variance as measures of error when extracting r = 2 through 30 signatures de novo using non-negative matrix factorization (NMF). Produced using the “assessNumberSignatures” function from the “SomaticSignatures” package [28]. Three replicates were run for each r value.

**Additional file 2: Figure S2**. Mutational signatures extracted de novo with NMF and r = 9 using the package “SomaticSignatures” [28].

**Additional file 3: Figure S3**. Mutational signatures extracted de novo with NMF and r = 4 using the package “SomaticSignatures” [28].

**Additional file 4: Figure S4**. Mutational signatures extracted de novo with NMF and r = 12 using the package “SomaticSignatures” [28].

**Additional file 5: Figure S5**. Mutational signatures extracted de novo with NMF and r = 20 using the package “SomaticSignatures” [28].

**Additional file 6: Table S1**. Comparison of COSMIC mutational signature contributions to non-seminomatous (n = 56) and seminomatous (n = 44) testicular tumors. Entries are number (proportion) of cases with a signature present above a threshold of 6% in each histologic group. *P*-values were calculated adjusting for age of onset * indicates statistical significance at a 0.05 level.

## Data Availability

The results published here are in whole or part based upon data generated by The Cancer Genome Atlas (TCGA) managed by the National Cancer Institute (NCI) and National Human Genome Research Institute (NHGRI). Information about TCGA can be found at http://cancergenome.nih.gov. The variant calls and clinical data analyzed during the current study are available through the Genomic Data Commons Data Portal, dbGaP accession number = phs000178, https://portal.gdc.cancer.gov/projects/TCGA-TGCT.

## Abbreviations

95% CI: 95% Confidence Interval
COSMIC: Catalogue Of Somatic Mutations In Cancer
DES: Diethylstilbestrol
GCNIS: Germ cell neoplasia in situ
RPMM: Recursively partitioned mixture model
SNV: Single nucleotide variant
TCGA: The Cancer Genome Atlas
VCF: Variant Call Format
