# Supplementary figures and images for: Mutational signatures among young-onset testicular cancers
Source: BMC Med Genomics. 2021 Nov 24;14:280. doi: 10.1186/s12920-021-01121-8 (PMC8611954; doi:10.1186/s12920-021-01121-8)

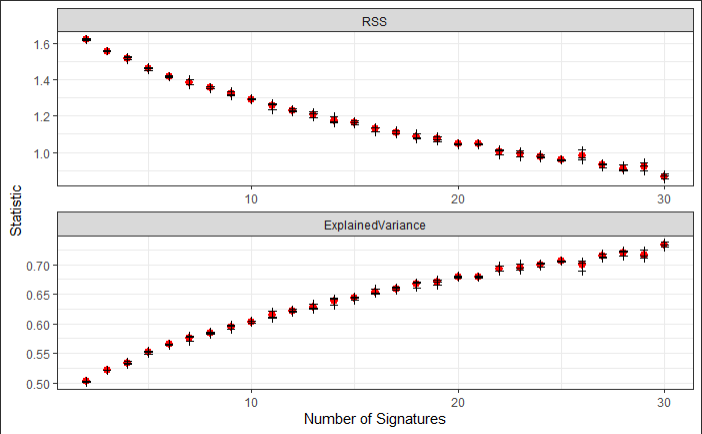

Supplement: Supplementary file 1 — Additional file 1: Figure S1. The residual sum of squares (RSS) and explained variance as measures of error when extracting r = 2 through 30 signatures de novo using non-negative matrix factorization (NMF). Produced using the “assessNumberSignatures” function from the “SomaticSignatures” package [28]. Three replicates were run for each r value. [file 12920_2021_1121_MOESM1_ESM.png]

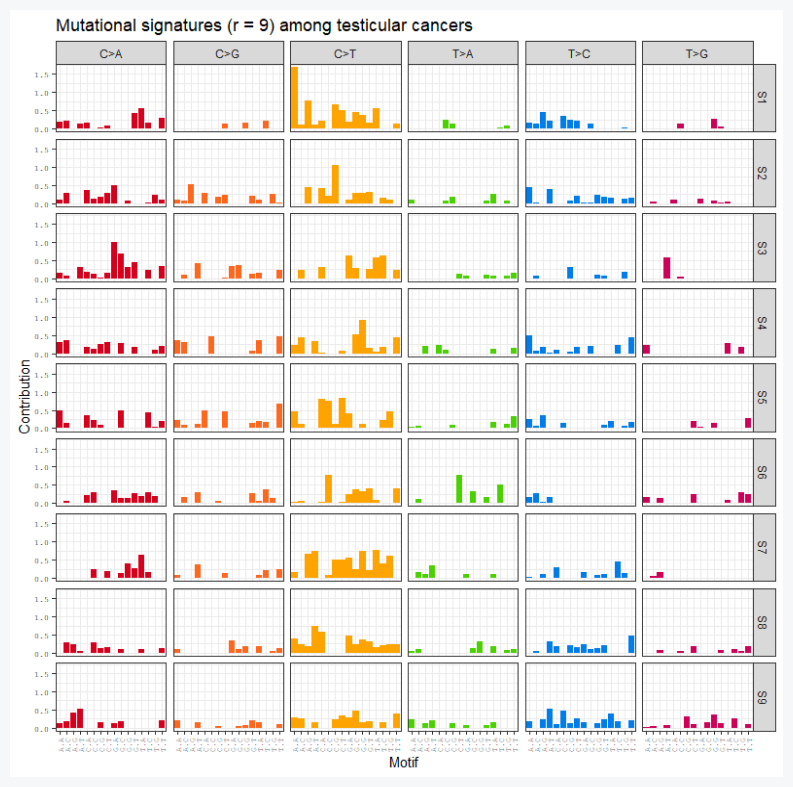

Supplement: Supplementary file 2 — Additional file 2: Figure S2. Mutational signatures extracted de novo with NMF and r = 9 using the package “SomaticSignatures” [28]. [file 12920_2021_1121_MOESM2_ESM.png]

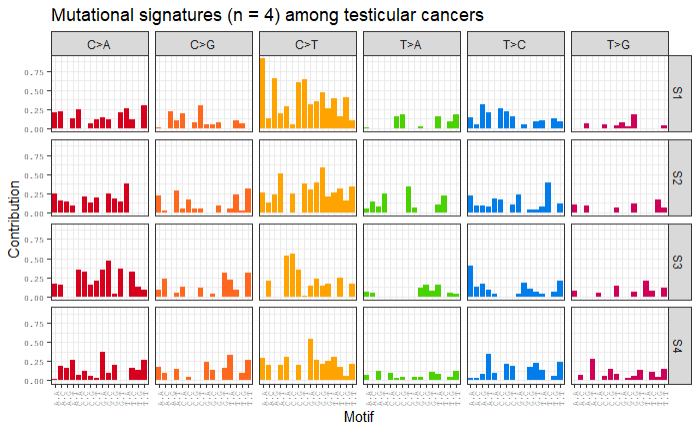

Supplement: Supplementary file 3 — Additional file 3: Figure S3. Mutational signatures extracted de novo with NMF and r = 4 using the package “SomaticSignatures” [28]. [file 12920_2021_1121_MOESM3_ESM.png]

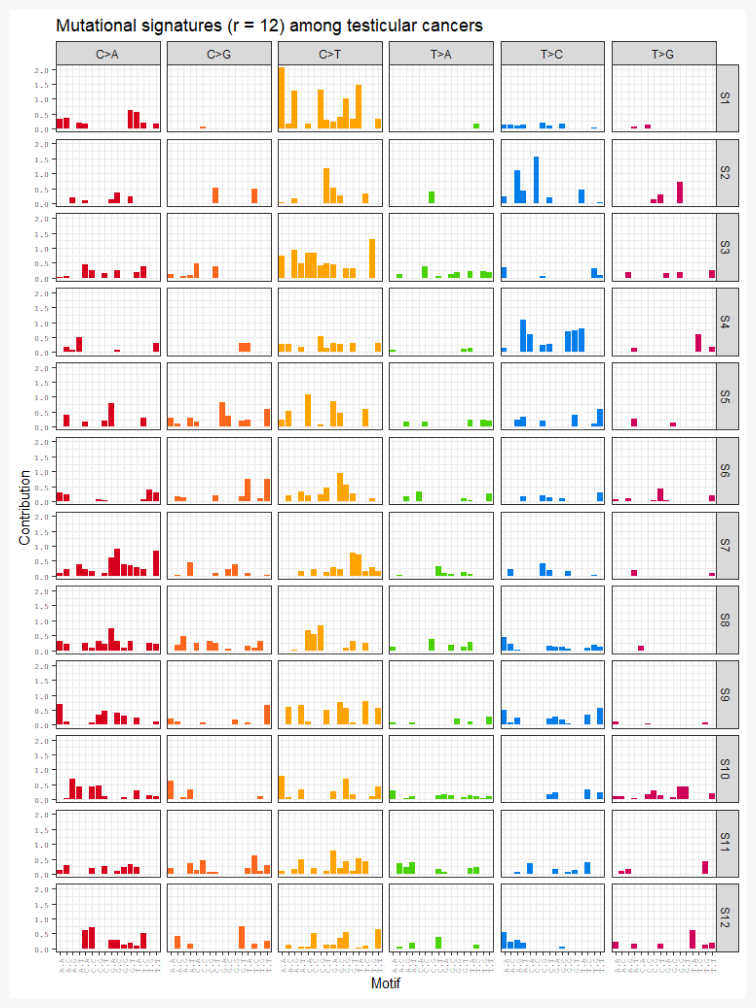

Supplement: Supplementary file 4 — Additional file 4: Figure S4. Mutational signatures extracted de novo with NMF and r = 12 using the package “SomaticSignatures” [28]. [file 12920_2021_1121_MOESM4_ESM.png]

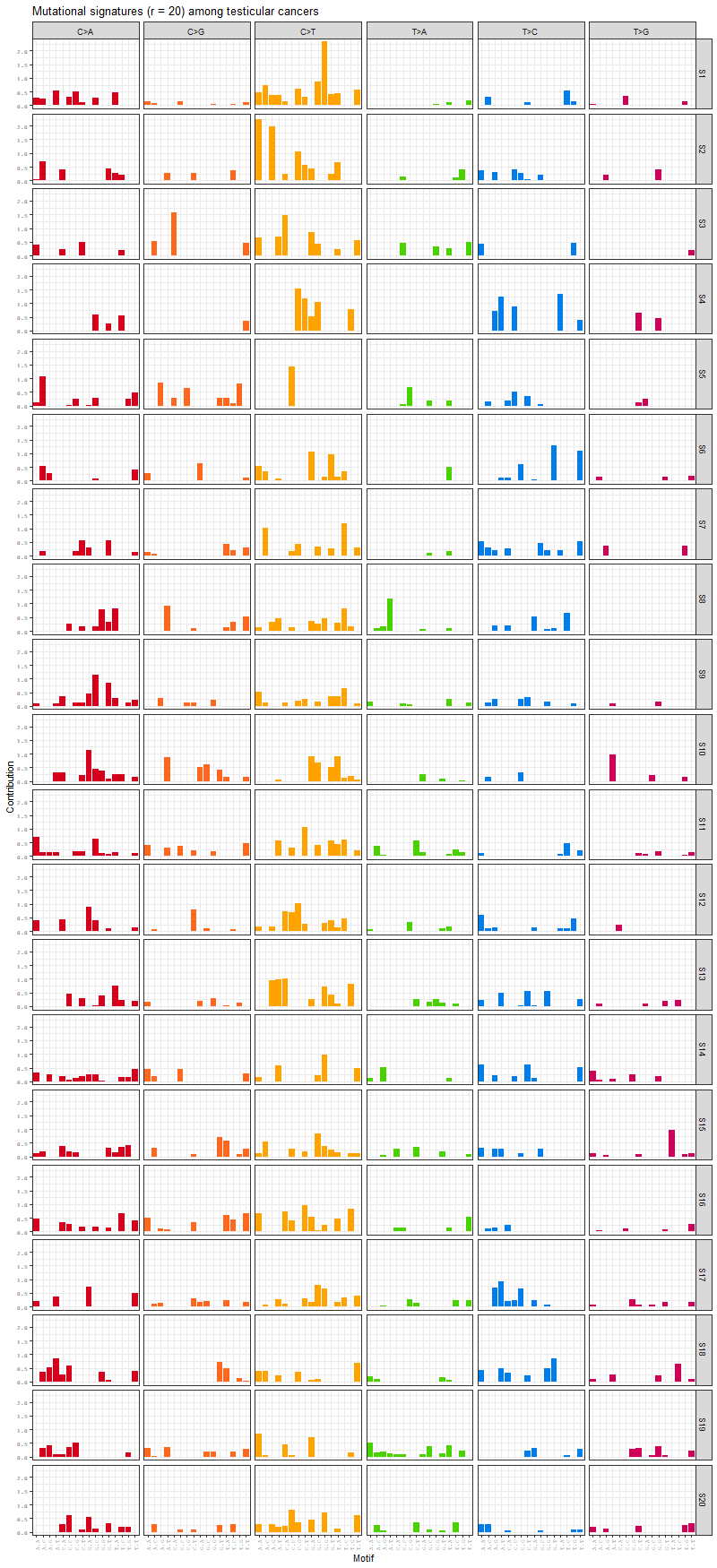

Supplement: Supplementary file 5 — Additional file 5: Figure S5. Mutational signatures extracted de novo with NMF and r = 20 using the package “SomaticSignatures” [28]. [file 12920_2021_1121_MOESM5_ESM.png]
